# WalkRollMap.org: Crowdsourcing barriers to mobility

**DOI:** 10.3389/fresc.2023.1023582

**Published:** 2023-03-15

**Authors:** Karen Laberee, Trisalyn Nelson, Darren Boss, Colin Ferster, Kate Hosford, Daniel Fuller, Marie-Soleil Cloutier, Meghan Winters

**Affiliations:** ^1^ Department of Geography, University of Victoria, Victoria, BC, Canada; ^2^ Spatial Pattern Analysis & Research Lab, Department of Geography, University of California Santa Barbara, Santa Barbara, CA, United States; ^3^ Cities, Health & Active Transportation Research Lab, Faculty of Health Sciences, Simon Fraser University, Burnaby, BC, Canada; ^4^ Department of Community Health and Epidemiology, College of Medicine, University of Saskatchewan, Saskatoon, SK, Canada; ^5^ Laboratoire Piétons et Espace Urbain, Centre Urbanisation Culture Société, Institut National de la Recherche Scientifique, Montréal, QC, Canada

**Keywords:** pedestrian, safety, comfort, mobility, hazards, barriers, crowdsourcing

## Abstract

Walking is a simple way to improve health through physical activity. Yet many people experience barriers to walking from a variety of physical, social, and psychological factors that impact their mobility. A challenge for managing and studying pedestrian environments is that barriers often occur at local scales (e.g., sidewalk features), yet such fine scale data on pedestrian facilities and experiences are often lacking or out of date. In response, our team developed WalkRollMap.org an online mapping tool that empowers communities by providing them with tools for crowdsourcing their own open data source. In this manuscript we highlight key functions of the tool, discuss initial approaches to community outreach, and share trends in reporting from the first nine months of operation. As of July 27, 2022, there have been 897 reports, of which 53% served to identify hazards, 34% missing amenities, and 14% incidents. The most frequently reported issues were related to sidewalks (15%), driver behavior (19%), and marked crosswalks (7%). The most common suggested amenities were sidewalks, marked crosswalks, connections (i.e., pathways between streets), and curb cuts. The most common types of incidents all included conflicts with vehicles. Data compiled through WalkRollMap.org offer unique potential for local and timely information on microscale barriers to mobility and are available for use by anyone as data are open and downloadable.

## 1. Introduction

Active transportation has tremendous potential to mitigate various societal issues, including but not limited to poor health from physical inactivity (1), greenhouse gas emissions from transportation (2), and social isolation (3). As few as 17% of adult Canadians meet the recommended 150 min per week of moderate to vigorous physical activity (4). Furthermore, people with disabilities are less likely to meet physical activity guidelines and generally have poorer health than the general population (5). Eliminating physical inactivity in Canada would reduce all-cause mortality by 9% and leading chronic diseases (cardiovascular disease, type 2 diabetes, and breast and colon cancers) by 6%–10% and increase life expectancy by an estimated 0.55 years (1). With easy integration into daily transportation routines, walking can help people increase their level of physical activity (6). However, many people do not live in neighborhoods where the built environment adequately supports walking (7), especially for older adults or people living with a disability (8).

Cities are setting ambitious goals to increase active transportation, including walking and rolling. Yet for many local governments, improving walkability remains a daunting task due to decades of planning that prioritized car travel over human-centered design (9). Many cities also lack current data on their existing pedestrian environments (10), especially at a higher spatial resolution. Roads, sidewalks, and crossing features may represent critical barriers that impact a person's ability to get around by walking or rolling. For example, the placement of hydro poles, street furniture, and even sandwich boards can create barriers for individuals using mobility devices (11). Less obvious barriers may include the lack of amenities such as benches or accessible washrooms. Other barriers may relate to personal safety concerns, especially for women or diverse genders (12). Increasingly, researchers are understanding the importance of identifying these barriers (13, 14) and capturing local nuances (15) when assessing the road and built environment—especially for vulnerable populations.

There are also gaps in official data sources for pedestrian injuries (e.g., falls, collisions from vehicles) across our communities. Falls as a pedestrian are a major public health concern, with data from the Netherlands suggesting that they are responsible for over half of all pedestrian fatalities and roughly 80% of pedestrian injuries requiring hospital admissions (16). Data on falls is primarily collected by hospital emergency departments without being georeferenced, limiting our understanding of the environmental circumstances that precipitated the injury. Further, many minor injuries from falls are not captured in official data, yet the resulting fear of falling, especially for older adults and people with physical disabilities, may restrict a person's mobility and contribute to poor health (17, 18). Injuries resulting from collisions between pedestrians and motor vehicles are also a serious health issue, with pedestrians accounting for 17.8% of road fatalities in Canada in 2019 (19). Many collisions involving pedestrians are not reported. Data from the UK's National Travel Survey suggest that as few as one in five “road accidents” involving pedestrians are accounted for in police data (20). Missing data from unreported collisions in police or public insurance databases hamper our ability to identify problematic locations and situations.

Thus, better data related to walking environments are needed to help communities meet the needs of those typically underserved by transportation systems including older adults, low-income people, racialized people, people with disabilities, children and women (21). As many communities around the world are experiencing an aging population, it is important to have data on minor pedestrian barriers that can be problematic as mobility declines. Barriers to walking for older adults often result from past falls or pedestrian environments that are poorly designed or maintained (22), such as sidewalks that aren't cleared after a snowfall. Improving pedestrian environments is also important for meeting goals of transportation equity, as lower income and racialized populations are more likely to walk in unsupportive environments, such as those with poor sidewalk infrastructure, and face a substantially higher risk of injury or fatality from a collision while walking (23). Finally, some barriers may relate to personal safety concerns, especially for racialized populations, Indigenous people, and for women and LGBTQ2S + people, who disproportionately experience crime, assault, harassment, and policing as they move through public spaces (12, 24, 25).

In the 2017 report, “Designing Healthy Living,” Canada's Chief Public Health Officer issued a call to action to re-design cities to support health—including improvements in data collection, explicitly considering context, and directly engaging cities (26). Crowdsourcing data provides an opportunity to fill in data gaps related to the urban built environment (27). For example, BikeMaps.org has provided rich self-reported data that have enabled a better understanding of the contextual issues at hot spot locations (28) and trends in crash scenarios across cities (29). Another crowdsourcing app, accessnow.com, rates the accessibility of public spaces such as restaurants, trails, and shops.

In this paper we introduce WalkRollMap.org—a web-based mapping platform that allows community members to map their microscale barriers to walking and rolling. We highlight key elements of the crowdsourcing tool, our approach to promoting the tool, and summarize trends in preliminary reporting. By focusing data collection on people's experiences while walking or rolling, we aim to capture and visualize the timely and nuanced local data that identifies barriers on routes that people use or would like to use and curate decision-making and action on pedestrian environments.

## 2. WalkRollMap.org tool

WalkRollMap.org is a web-map where people can report pedestrian hazards or concerns, suggest missing amenities, or report a collision, near miss, or fall. WalkRollMap.org is loosely based on an earlier crowdsourced tool developed by our team, BikeMaps.org, which gathers crowdsourced reports of bicycling collisions and near misses (30). We used our experience building, promoting, and maintaining BikeMaps.Org to inform the development and design of WalkRollMap.org.

**Figure 1:**
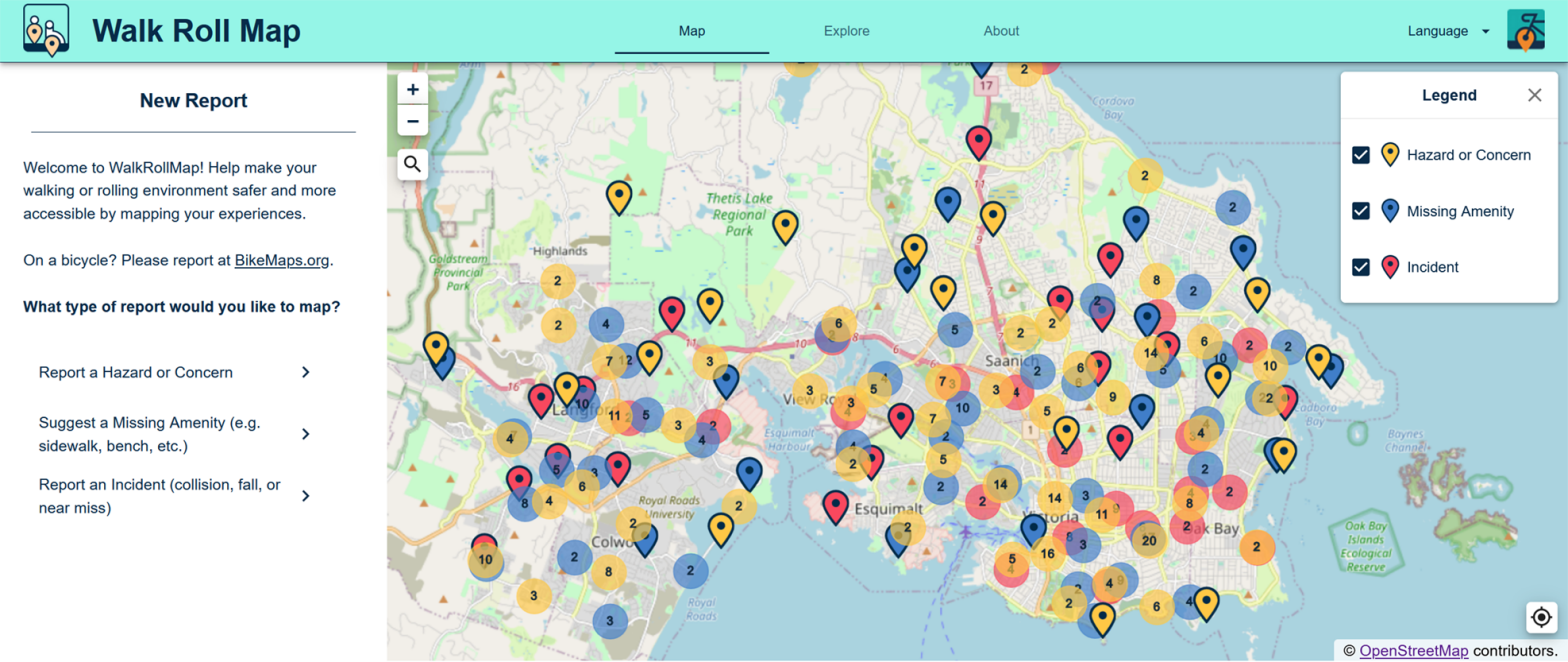
Landing page for WalkRollMap.org.

WalkRollMap.org was built with free and open-source tools. The website is welcoming: the community mapper sees a Google Maps-like interface (Figure 1), although the map technology used is OpenLayers 6, a JavaScript mapping library that can display map tiles, vector data, and markers loaded from a wide variety of sources. The website front-end is a single page application built with React, a declarative, component-based JavaScript framework that enables the creation of interactive user interfaces. We designed the front-end with a mobile first approach and followed responsive design principles, so that the website can be viewed on a variety of devices (desktop computer, laptop computer, tablets, and mobile phones) following accessibility guidelines (w3.org/WAI). We used additional opensource JavaScript packages that enhance functionality, provide professional styling, dynamic user interaction, and components for rendering map content. The database system is PostgreSQL coupled with the PostGIS extension; together they accommodate efficient storage and querying of spatial data. The back-end web services are based on Flask, a micro web framework written in Python. The Flask application exposes a REST API that allows for the two-way flow of data between the database and front-end website. The website is available in English, French and Spanish.

To determine what barriers the survey should capture, we reviewed the literature on microscale barriers to walking or rolling and conducted semi-structured interviews with key stakeholder informants. Interviews were conducted over Zoom between December 14, 2020 and March 8, 2021 with 24 participants from across Canada: Greater Victoria (*n* = 11), St. John's (*n* = 5), Ottawa (*n* = 4), Vancouver (*n* = 2), and Ajax (*n* = 1). Participants included pedestrian advocates (*n* = 7), city staff (*n* = 6), elected officials (*n* = 4), people who face mobility challenges (*n* = 4), a parent of a child who uses a wheelchair (*n* = 1), and a city consultant (*n* = 1). A semi-structured interview guide was used, which we adapted to the participants' lived experience or role regarding pedestrian environments. Interviews lasted between 40 and 60 min in duration, were audio-recorded, and professionally transcribed. Themes were initially coded by co-author KH in NVivo and discussed with team members. Specific themes related to barriers fell into eight categories and are included with common examples in Table 1. Some barriers cited were specific to certain population groups. For example, unpredictable snow clearing was a barrier for a person with a vision impairment and a bumpy road surface made it very uncomfortable for a person who uses a wheelchair.

**Table 1:** Main categories of barriers from the stakeholder interviews.

Category of Barrier	Examples
Infrastructure	Missing or inadequate sidewalks, crosswalks, surface conditions, and intersection infrastructure (e.g., curb cuts, audibles).
Missing Amenities or Features	Benches, washrooms, poor or no lighting, wayfinding signs, planters/vegetation
Pedestrian Network	Missing trail connections and cut throughs
Obstructions	Garbage/recycling bins, bollards, tripping hazards, poles, sandwich boards, construction, e-scooters, and bicycles
Road Safety	Speeding vehicles, poor sightlines, parking, unsafe intersections
Personal Safety	Poor lighting, crime, harassment
Other users	Bicycles, vehicles
Environmental	Snow, ice, splash zone

**Figure 2:**
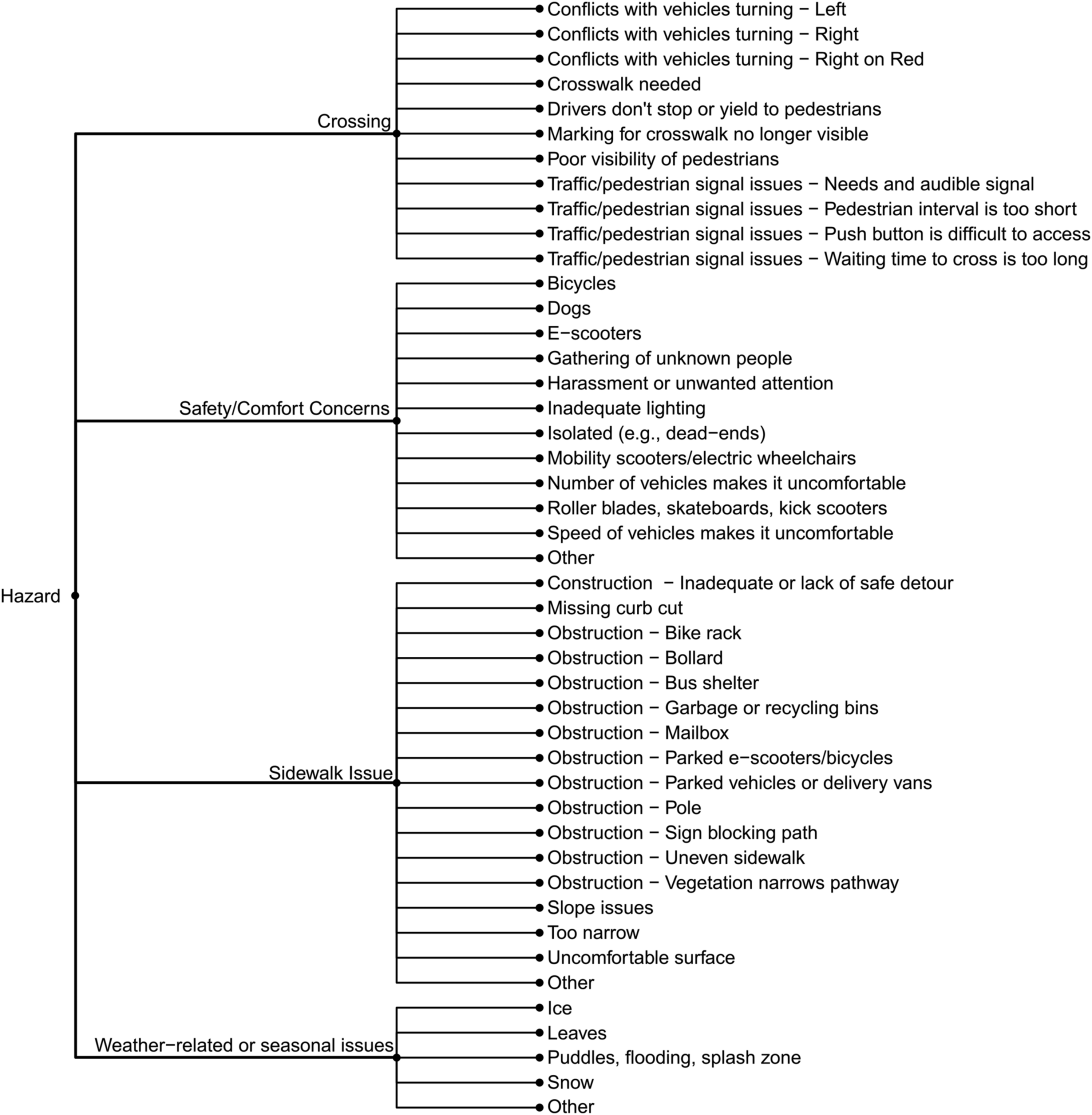
Choice of nested responses if participant chooses to map a Hazard or Concern.

**Figure 3:**
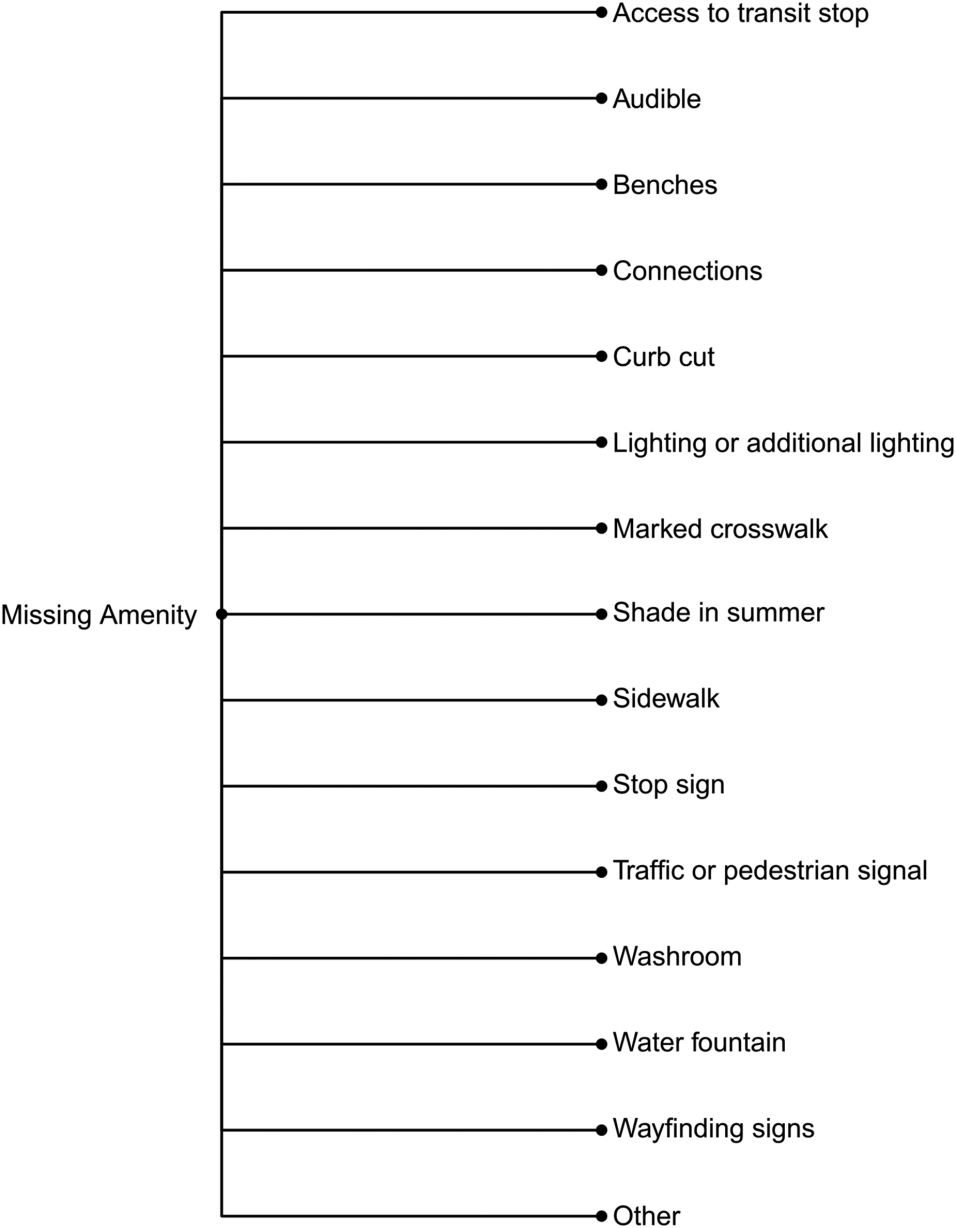
Choice of responses if participant chooses to map a Missing Amenity.

**Figure 4:**
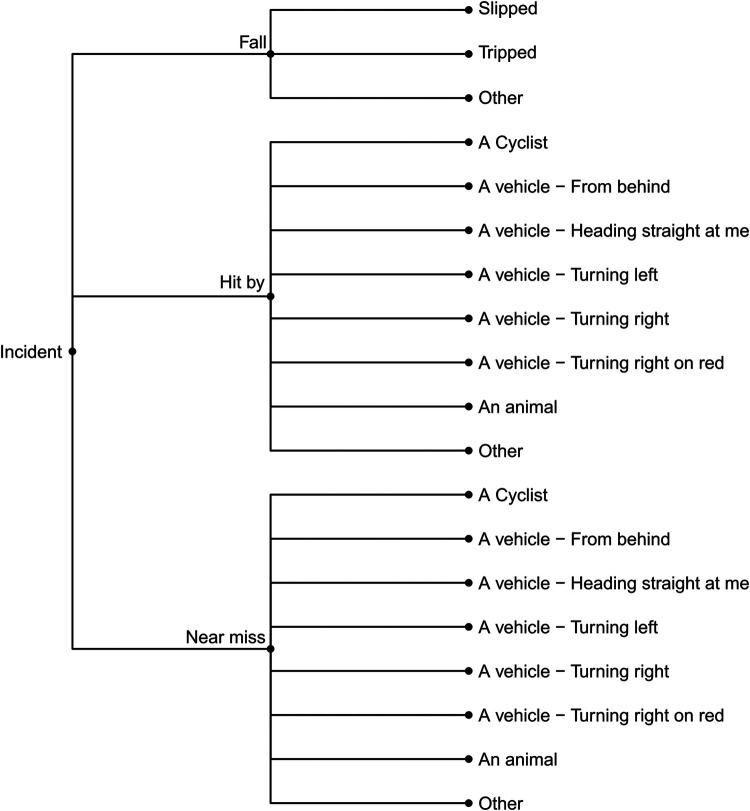
Choice of responses if participant chooses to report an incident.

**Figure 5:**
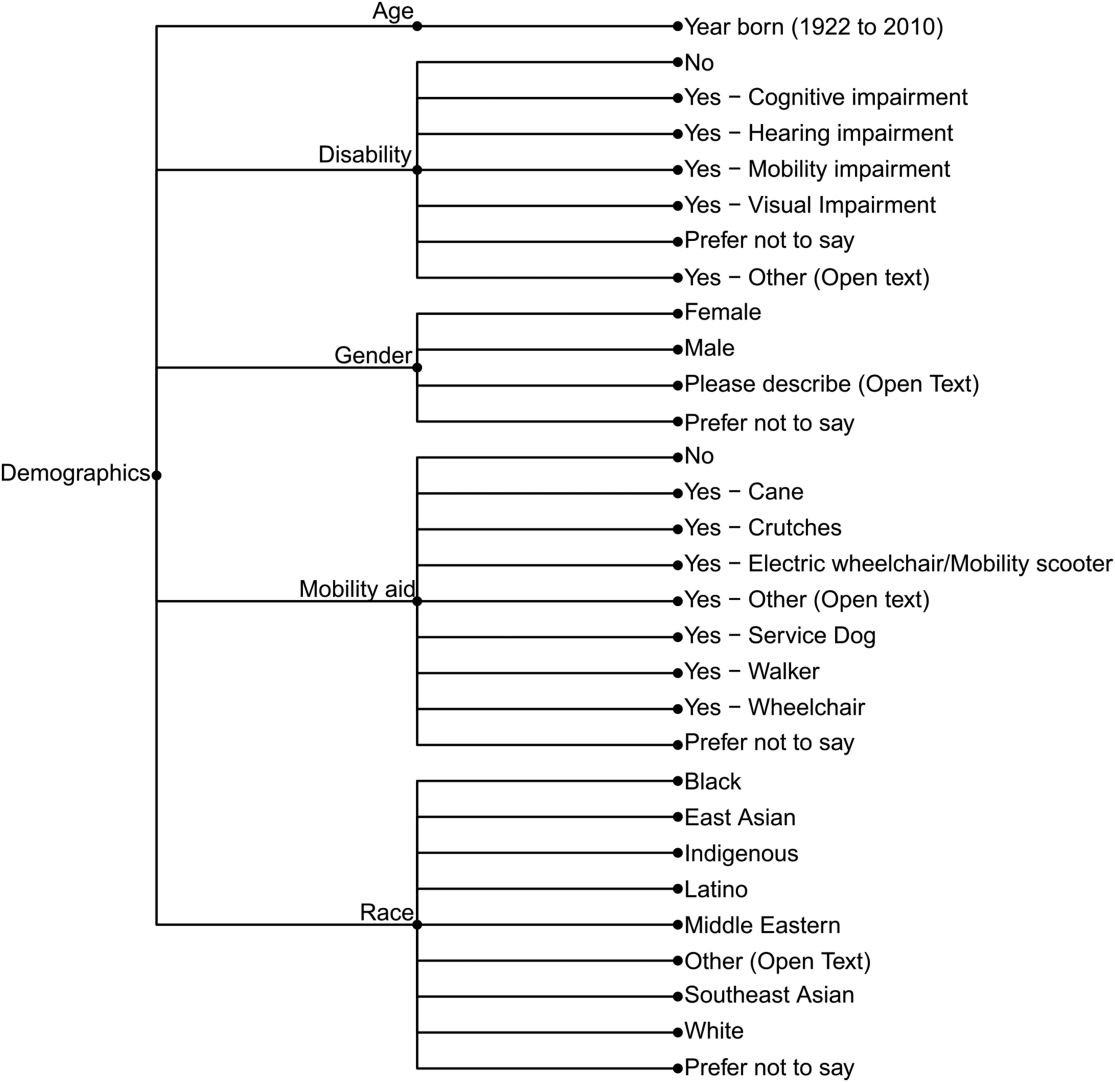
Demographic data collected on WalkRollMap.org survey.

We ultimately organized the survey questions such that community mappers have the option of three broad categories of reports: (1) Hazards or concerns, (2) Missing amenities, and (3) Incidents (falls, near misses, or collisions). The subcategories of hazards or concerns are detailed in Figure 2 and those related to missing amenities in Figure 3. Figure 4 outlines the data collected for an incident (collision, fall, or near miss). For each incident we asked if the incident (i) happened to the person reporting; (ii) happened to someone in their care; or (iii) they witnessed the incident. In addition to collecting data on the hazard or concern, missing amenity, or incident, we ask demographic questions to determine gender, age, race, and any mobility issues on all reports (Figure 5). Finally, we included a mandatory open-ended description field for the participant to provide additional details on their report as our work on BikeMaps.org has shown that these narratives provide valuable context for informing research or action (28, 29).

**Figure 6:**
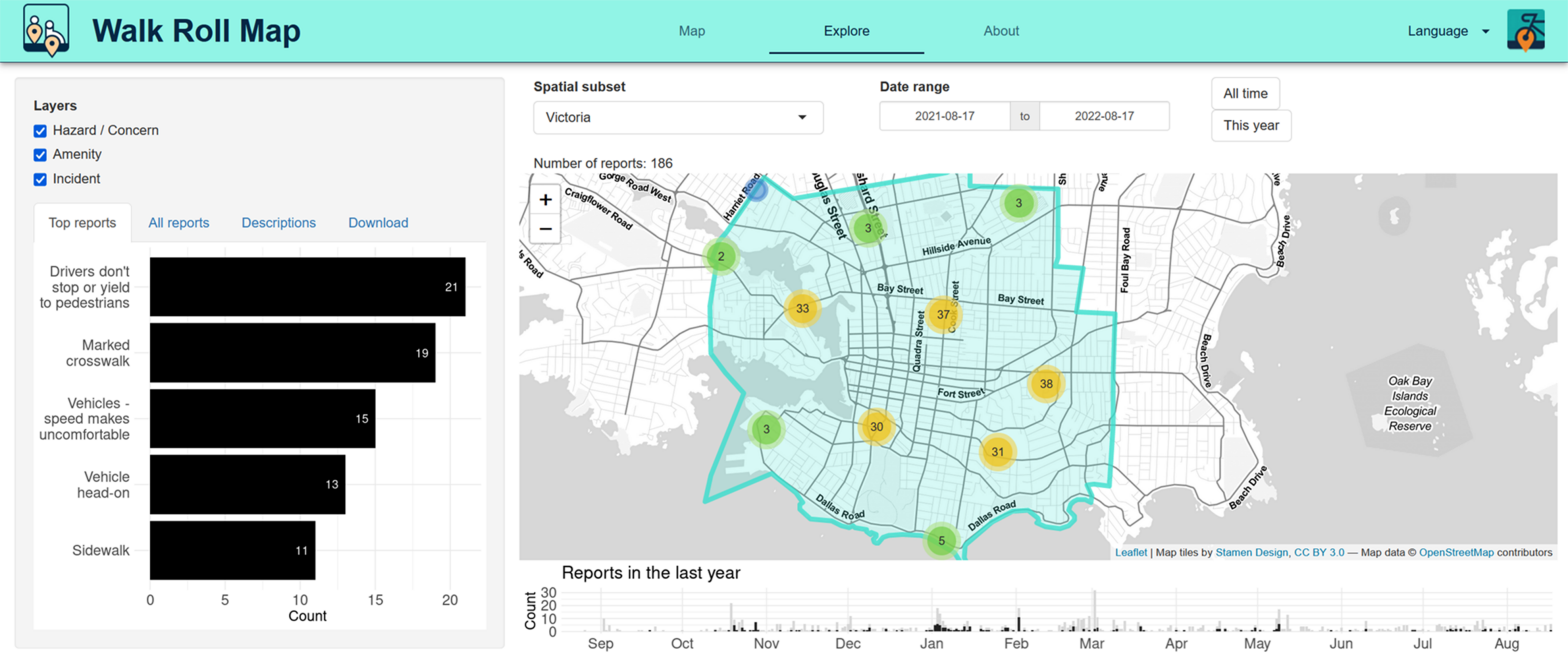
Example of visualization of WalkRollMap.org data for Victoria, British Columbia, Canada.

WalkRollMap.org includes a tool for visualizing patterns in the data collected (Figure 6). Available in the explore tab, the visualization tool allows filtering of reports by type of report, date, and location (map extent or city boundaries for collaborating municipalities). The visualization tool provides a view of the data on a map, a timeline of reports, a bar chart of top issues reported, a detailed treemap of all issues reported, a word cloud of the text descriptions of the issues, and the ability to download the filtered data in plain text (comma-separated values) or spatial (keyhole markup language or geopackage) formats. The app was developed as a Shiny App in R.

Using the visualization page, for example, it is possible to determine the most common types of reports. To date, missing sidewalks, reports of uncomfortable vehicle speeds, and missing marked crosswalks are the most commonly reported barriers. However, this changes by region, which can be adjusted by panning in and out of the map. Also, by simply clicking on a feature one can filter hazards, incidents, and missing amenities to see the top reported concern in each data category, as well as the top reports overall. A user can also query reports by date. Filtering by date is particularly important for seasonal concerns, such as snow and leaves on the ground. The visualization tool also makes a word cloud diagram from the open-ended text descriptions to highlight keywords used to describe concerns. For example, when focused on western North America, the word diagram highlights the words: road, cross, traffic, crosswalk, traffic, and access, indicating that these are the key concerns for people reporting to WalkRollMap.org.

## 3. Early promotion and data submissions

We publicly launched the English version of WalkRollMap.org in October 2021. Our focus was on promotion across our pilot region which was the City of Victoria and surrounding Capital Regional District of British Columbia, Canada (CRD). We shared the project through the BikeMaps.org blog and Twitter accounts associated with the team or partners. To promote the platform with the public we hosted a table at three community events and attended several community walks for older adults. The rapid rise in the Omicron variant of COVID-19 in December 2021 curtailed in-person events and we pivoted to online promotion *via* Facebook ads in January 2022. With COVID-19 restrictions easing in late winter 2022, we found opportunities for outreach including hosting tables at community events and organizing walks.

As of July 27, 2022, there have been 897 reports made globally. Most of these reports are from the Capital Region (*n* = 640), where we focused our promotion efforts. The remaining reports are from other cities in North America (*n* = 244), Australia (*n* = 2), and Europe (*n* = 4).

Of the 897 reports, 37% are from men, 52% from women, and 2% identify as non-binary. In terms of age, a third (33%) of reports were from people aged 35–44 years old, a quarter (28%) from those 35 years or less, and just under two-fifths (39%) from those 45 years or older. Ten percent of reports were made by people who reported a disability, and 7% of reports were made by people who reported using a mobility aid. The most common type of disability was mobility (6 of all reports—consistent with the reporting of mobility aids), and the most common mobility aid types were canes, crutches, or walkers (3% of all reports), followed by wheelchairs (2% of all reports).

To monitor representation of our reach, we compare the demographics of respondents in the CRD to the 2016 Canadian census (Table 2). The average age of WalkRollMap.org respondents in the CRD was 43.3 years while the average age in the census was 44.4 years. 55.5% of reports were made by people identifying as women in the CRD compared to 51.8% in the census. To estimate racial representation in WalkRollMap.org reports compared to the broader population reported in the 2016 census (the most recent available census data), we used race categories from Winters et al. (31). For WalkRollMap.org data, the race categories are not mutually exclusive, so for example, a person who reported their race as white/East Asian would be counted in both categories, and as a result, the totals sum to greater than 100%. For 2016 census data, we used the Minority/Origin variable where the categories are mutually exclusive. Following Winters et al. (31), we estimated the percentage of population as white from the population that is not part of a visible minority minus the proportion of people with North American aboriginal origins. Compared to census data in the Capital Regional District in 2016, there were more reports by Latinx, women and older people. Indigenous and East Asian people were underrepresented and will require additional effort on our part to reach more effectively.

**Table 2:** Comparison of walkRollMap.org self-reported age, gender, and race with the 2016 Canadian census for the capital regional district (BC).

Race	WalkRollMap.org Reported (%)*	2016 Census (%)
White	75.5	80.2
East Asian	3.5	8.0
South Asian	3.4	2.8
Indigenous	3.2	6.1
Black	0.6	0.9
Middle Eastern	0.9	0.7
Latinx	2.1	0.7
Other	3.0	0.5
No response	16.1	NA
Gender		
Woman	55.5	51.8
Man	32.8	48.2
Other	0.9	NA
No response	10.8	NA
Age		
Average	43.3	44.4

*Please note that more than one race may be reported on WalkRollMap.org. Race categories from census data are derived from Minority/Origin categories and are mutually exclusive (e.g., sum to 100%) whereas WalkRollMap.org data allows multiple race responses per individual.

Of the 897 reports, 471 (53%) were hazards, 303 (34%) were missing amenities, and 123 (14%) were incidents. The five most common hazards were: (1) drivers who do not stop or yield to pedestrians, (2) vehicles—speed of vehicle makes me uncomfortable, (3) safety/comfort concern (other), (4) obstruction—uneven sidewalk; and (5) poor visibility of pedestrians. The five most common missing amenities were: sidewalk, marked crosswalk, connections (e.g., cut through or pathway between streets needed), benches, curb cut. The five most common incidents all included conflicts with vehicles moving in a variety of directions (heading straight, turning left, turning right, from behind, and turning right on red).

## 4. Discussion

The purpose of this paper is to present WalkRollMap.org and offer insights from our processes of designing, using, and promoting the tool. In their scoping review of the factors affecting the ways in which people with disabilities access walking or wheeling, Prescott et al. assert that municipalities need to better understand the current state of their pedestrian infrastructure (32). As such, many cities are investing in open GIS data, including extensive data on transportation infrastructure like roads and sidewalks. Yet data on the microscale features that are so important for pedestrians are difficult to keep up to date. Further, data on features such as park benches, crosswalks, crime, lighting, and water fountains, are often managed by different departments. Weather-related elements and other transient issues such as construction work may also pose barriers and contribute additional risk factors. WalkRollMap.org helps to address these data gaps by providing a tool for crowdsourcing the microscale barriers to walking or rolling and pedestrian incident data. By empowering communities to build their own crowdsourcing pedestrian maps, our tool enables the integration of diverse perspectives, infrastructure data and pedestrian behavior data into a single database. Our platform allows barriers—both transient and permanent—to be mapped anytime and anywhere, therefore supporting stakeholders who want to tackle those barriers and work towards more equitable and healthy cities.

Our preliminary results point out hazards, amenities and incidents that are in line with the existing literature, proving the relevance of such a tool, especially for vulnerable populations. For example, most common hazards and sources of incidents reported on WalkRollMap.org are related to drivers within moving vehicles (e.g., drivers not yielding, or moving at speeds that cause pedestrian discomfort), which has also been reported in other studies on pedestrians' perception of walkability and safety (33). Other early results on WalkRollMap.org include concerns about uneven or missing sidewalks and missing benches, curb cuts, and connections. Previous research indicates that hazards and missing amenities increase the risk of falls and fatigue, especially for seniors and people with disabilities (20, 34), and reduce the willingness to walk among adults and children (35). Future analysis of data collected on WalkRollMap.org during all the different seasons should help identify other types of barriers. For example, the presence of snow or ice on the walking surface can more than double the risk of falling (36) and although temporary, can present extremely difficult barriers to mobility for some people (e.g., those who use wheelchairs and cannot step over snow windrows). Similarly, issues related to women's safety in public space were not mentioned as primary concern but might arise when more reports will be available since it is well-established that a fear of violence shapes women's everyday movement in public space (12, 37, 38). Having better data on this specific topic at the microscale through WalkRollMap.org could help authorities in their actions to reduce the level of harassment and violence in public space.

While our preliminary results offer valuable insights into how we can collect data about pedestrian environments, challenges remain. Unlike cyclists using BikeMaps.org, we have found it more difficult to mobilize people to report barriers to walking and rolling. This may be because walking is so widespread that few people identify as a pedestrian and fewer still as a pedestrian advocate. Walking is just something we all do. With BikeMaps.org, we have found that an effective way to motivate people to take time to contribute their data is to have a clear pathway for how the data will be used either in a specific research project or with a community partner and we will need to do the same with WalkRollMap.org. Second, gathering demographic data on who is mapping is important for monitoring the project's outreach efficacy and better understanding how barriers may be felt differently depending on age, race, gender, income, or disability. However, representativeness of data is often a challenge when data are collected through crowdsourcing. Data will reflect the experiences of the population that contributes, which tends to be people who already have access to technology and transportation choices (30). In this project we have given data representation extensive attention. For example, our team led walks with older adults to facilitate their usage of the application and, in turn, their data contributions. Yet, some groups are still underrepresented in data contributions and efforts are being made to improve diversity in sampling through outreach and by building local, effective partnerships to increase data representativeness.

As people continue to rediscover walking for health, enjoyment, and/or transportation after a prolonged period of pandemic-related sedentary behavior, some will need to navigate pedestrian environments that are not designed with their presence and diversity in mind (e.g., pedestrian spaces not designed for those living with mobility impairments, or the rest/break requirements for older people). Through gathering crowdsourced data on WalkRollMap.org we hope to enable many communities to document barriers to safe and comfortable walking or rolling in order to effect change locally. As we expand our outreach efforts into new communities, we will continue to put extra effort into reaching priority populations to capture the lived experiences of those who are most impacted by poor pedestrian environments. These data will not only serve as a resource for municipalities looking to plan or prioritize active transportation but will offer researchers insight into the range of barriers that prevent people from simply *walking places*.

## Data Availability

The datasets presented in this study can be found in online repositories. The names of the repository/repositories and accession number(s) can be found below: Data is available at WalkRollMap.org/about.
